# Parents’ Autistic Personality Traits and Sex-Biased Family Ratio Determine the Amount of Technical Toy Choice

**DOI:** 10.3389/fpsyg.2019.02101

**Published:** 2019-09-20

**Authors:** Chris Lange-Küttner, Messiah A. Korte, Christina Stamouli

**Affiliations:** School of Social Sciences, London Metropolitan University, London, United Kingdom

**Keywords:** parenting, autism quotient (AQ), toy choice, mere presence effects, offspring sex

## Abstract

We investigated the effect of parents’ autism quotient (AQ), their sex and the sex of their children on their toy preference. In a computerized forced-choice shopping task, adults selected from cuddly and social role-playing toys (social toys), academic, music and sports toys (educational toys) and construction sets as well as cars (technical toys). A sex-balanced high and low AQ sample of 160 adults consisted of groups of parents of sons only, daughters only, sons and daughters, as well as of a group of adults without children (non-parents). The standard toy preference was social toys < educational toys < technical toys. Low AQ women were the only group to make a significantly higher amount of educational and a lower amount of technical toy choices. The mere presence of just sons increased technical toy choice in this experiment, while the mere presence of just daughters reduced technical toy choice both in men and high AQ individuals.

## Introduction

Autism in children is seen as a strongly genetic disorder (e.g., Bailey et al., 1995; Grove et al., 2019). However, it is also generally agreed that there is an autistic spectrum where symptoms of repetitive behavior, lack or diminished language development and communication as well as empathy are personality features that are less pronounced and amount to an autistic phenotype rather than a clinical diagnosis (Bailey et al., 1998; Grofer Klinger and Dudley, 2019). Nevertheless, integration of different perspectives on the autistic spectrum disorder (ASD) have been called for in the past (Bailey et al., 1996). One of the striking features of autism in children mentioned already in one of the first published studies (Asperger, 1944) was that their parents were not from deprived areas, but fathers in the majority were from technical professions, that is engineers or electrical engineers (Hippler and Klicpera, 2003). In contrast, most of the fathers in the control group of children who attended Asperger’s clinic for other reasons such as behavioral problems and attention difficulties were manual workers. Similarities in personality between parents and their autistic child were also noted, e.g., being aloof or ‘nervous.’

We did not ask the parents in this study about their parental professional (engineering) choices, but used a toy choice paradigm – which involved technical toys – in order to investigate whether autistic traits would also translate into the choices parents make for their offspring. The hypothesis was that parents of either gender who score low on sociability and high on autistic traits would be more likely to select technical toys for their children. To compare, we used three categories of toys, technical, social, and educational toys.

We investigated parents’ toy choice with an experimental toy choice task using a stratified sample of parents who showed low or higher autism quotient (AQ) sub-clinical scores in the AQ questionnaire (Baron-Cohen et al., 2001). Toy choices are important in play therapy and can help children overcome problematic behaviors and regulate their emotions (Reinhartsen et al., 2002; Laue, 2016). The current study, however, shows in addition that the mere presence of just daughters can help their parents overcome some limitations of their interests resulting from their personality.

### The Toy Choice Paradigm

Historically, the first research studies on toy choice focused on age and sex differences (Benjamin, 1932; Kawin, 1938). Benjamin found that boys preferred to play with cars, while girls preferred to play with dolls. Toys such as an aeroplane or a horse were more or less preferred by both sexes. This line of research has been continued until today. A recent meta-analysis of 1788 papers with a final sample of 787 boys and 813 girls showed that the results of the early studies stayed remarkably robust especially for boys, so much so that a biological origin is assumed (Todd et al., 2017). However, sex of children was predictive for gender-typed play over and above the biological variable amniotic testosterone (Knickmeyer et al., 2005). While traditionally the term ‘gender’ is used for the belief that differences between males and females are culturally determined, the term ‘sex’ is used for the belief that these differences are biologically determined. We believe that the difference should be treated just as a statistical variable (Lange-Küttner, 2017) because there is considerable within-group variation within groups of males and females, respectively, depending on context.

It was also asked whether toy choice preference of children is actually shaped by their parents. For instance, well-educated mothers report that they would look for toys that are hygienic, ecological, well-designed and safe, and mothers from a better socio-economic background would in addition monitor that the toy is attractive to the child and that it would support the development of various developmental domains (Kabadayı, 2014).

Other researchers investigated whether the toy choice of children is influenced by their parents’ sex role preferences. One study reported an overlap of 81–91% between parents and children when it came to sex-specific toys (Peretti and Sydney, 1984). Children at age 4–5 tend to conform with parents’ expectations that toys which apparently do not conform with their own sex are bad (Raag and Rackliff, 1998; Raag, 1999; Kollmayer et al., 2018). Parents may hold conservative sex-specific concepts of toy choice, but in the actual play situation, they spent the least time with feminine toys (Idle et al., 1993). Also in another study of 1- to 5-year-old children’s spontaneous play – with or without a parent – feminine toys such as feeding bottles and beauty sets became less interesting for both boys and girls with increasing age (Servin et al., 1999).

But even if the parents disapproved of common sex stereotypes, their children predicted that their parents would judge their toy choices in this way (Idle et al., 1993). Still even these less prejudiced parents were less comfortable with boys’ expressive behavior (‘boys don’t cry’) than with assertive girls who were tomboys. This may reflect parents’ concern that their offspring will be able to achieve in a competitive society in the future.

However, there was a subtle difference when children would judge toys for themselves versus others. While children attribute sex-specific toy choice preferences to other children, for themselves, they were mostly interested on what the toy could do, that is, they had a rather functional view on toy choice (Eisenberg-Berg et al., 1982). Together, these results reflect some deep-rooted belief about which toy is appropriate (Lobel and Menashri, 1993; Zimmermann, 2017) which appear to co-exist with a rather practical approach in the actual play situation.

In an in-depth study that considers both the behavioral factors as well as the attitudes of parents, mothers’ ‘femininity’ (expressiveness) and fathers’ ‘masculinity’ (assertiveness) (Turner and Gervai, 1995, p. 764) had no significant effect on children’s toy preference. Instead, fathers who showed more participation in the household shaped more typically female play activities, fathers’ expressiveness predicted less typically male peer interactions and their assertiveness predicted fewer typically female peer interactions. Only mothers with more traditional role models predicted more typically male toy preferences. Interestingly, fathers of daughters were more expressive and less sex biased in their childrearing attitudes then fathers of sons, while mothers’ scores were not influenced by the sex of their children.

### The Autism Quotient (AQ) in Parents

We investigated whether the personality of the parents had an impact on toy choice. Different to the IQ (intelligence quotient), the AQ measures sociability, that is a more detached attitude toward social activities with people, with a focus on systematic analyses which is more common in men particularly in the STEM professions (science, technology, engineering, mathematics) (Baron-Cohen et al., 2001; Wei et al., 2013), see also the more detailed description of the AQ questionnaire in the “Materials and Methods.”

According to a recent review (Ruzich et al., 2015a, b) with a total sample of *N* = 1,374, the non-clinical adult population has an average AQ of 17, while the clinical population has a score of 35. Mentally healthy male participants tend to score higher on the AQ questionnaire than females (18:15), while in clinical samples the AQ score sex ratio was reversed to 36:39. When individuals with a milder form of autism as per Broad Autism Phenotype Questionnaire (BAPQ) were excluded, this sex ratio was still 15:13 showing that in clinical samples, AQ in women is not less pronounced than in men. Individuals who score high on the BAPQ show an aloof and rigid personality as well as pragmatic language deficits with impaired or limited conversation (Hurley et al., 2007).

Hence, when individuals with a high AQ become parents, we hypothesized that their preference may be to select predominately technical toys for their children. The influence of parents on children’s toy choice is often seen as a cultural preference (e.g., Kollmayer et al., 2018), but parents’ toy preference may be more determined by their personality than researchers often assume.

Offspring of fathers in STEM professions more often show symptoms of autism than in the general population (Baron-Cohen, 1998; Roelfsema et al., 2012). Moreover, fathers of high-functioning autistic children responded slower to social cues (Scheeren and Stauder, 2008) although in this study, both the 25 fathers of typically developing children and those 25 with children on the autistic spectrum did neither differ on the IQ sub-test block design, nor in their AQ score. Also in another recent study with a much larger sample of 299 families with autistic children and 274 families with typically developing children, broad phenotype autism (BAP) features of the fathers were associated with autistic children’s social responsiveness, while mothers’ BAP scores were associated with the social responsiveness of the typically developing children (Shi et al., 2015). Moreover, parents of autistic children were less likely to use language for their understanding of others’ facial expression and emotions in comparison to parents of typically developing children (Gokcen et al., 2009). Also in this study, fathers were less adept in recognizing emotion independently of whether they had autistic children, or not. Given such effects of less sociably adept fathers, we investigate whether parents’ AQ score has an impact on their toy selection. In particular, we predict that parents with a high AQ score will select more technical toys, and parents with a low AQ will select more social role-playing toys. Because both mothers and fathers can have a high AQ score, we predict that AQ will have an effect on toy selection independently of the sex of the parent.

### The Emotional Family Climate

The second research question that we have is whether parents in families where all members are male with the exception of the mother would have different toy preferences than parents from families where all family members are female and the father is the only male person (gender-biased family ratio).

While there is an extensive literature on gender balance in the professions (e.g., Peeters et al., 2015; Haynes, 2017), we could not find a comparable amount of psychological studies on gender balance in families. One exceptional study by Campenni (1999) had used the same categorization in a study on gender stereotyping of children’s toys and also included a non-parent control group. She found that the daily interactions in female-majority households increased gender stereotypes in the fathers.

It is known, though, that especially Indian (Diamond-Smith and Bishai, 2015) and Chinese families (Sun and Zhao, 2016) prefer to raise sons over daughters even if they live in a Western country such as the United States (Almond and Sun, 2017). Sex-biased child rearing was also prevalent in LGBT families, with male-biased selection in adoptive families with two fathers and female-biased selection in adoptive families with two mothers, but mixed-sex adoption in heterosexual families (Golombok et al., 2014). These results show that the mere presence of sons, or the mere presence of daughters can be preferred, nevertheless, with the exception of adoptive families, it is nature that determines whether parents will have just sons, just daughters, or both sons and daughters, and parents have no choice in that matter.

The gender of the children in the family can indeed have a selective effect on a parent. For instance, in families of Australian Vietnam veterans, PTSD and depression of the father negatively impacted on the family emotional climate. When the family had a secure attachment to the veteran, PTSD symptoms were low, but symptoms were highest with inconsistent attachments to both sons and daughters. In contrast, symptom severity was not related to the attachment with the wife which shows the significance of the children. Moreover, the impact of the veteran’s PTSD correlated with the daughter’s, but not the son’s perception of the family emotional climate (O’Toole et al., 2018). This study thus shows that a father in a gender-mixed family appears to take his cues from the daughter. In another study, avoidant communication with the father predicted the role of a cyber-bullying victim for an offspring, while open communication with the mother prevented it (Buelga et al., 2017). A similar family pattern was found for sons and daughters of fathers with an alcohol problem (Tweed and Ryff, 1996).

Thus, while none of these studies controlled whether families were in the majority male, female, or mixed, they show the important effect of females for social functioning and mental health in the family, whether mother or daughters, for better, but also for worse: For instance, while parental autistic symptoms lead to a negative effect on family relationships, a mother with attention-disorder hyperactivity symptoms was likely to lead to family disruption (van Steijn et al., 2015).

Most studies assess family climate in gender-neutral ways on factors such as cohesion and adaptability (e.g., Sharabi et al., 2012). However, we assume that the gender balance in the family plays a role. If the father is the only man in the family, and there are at least two women (wife and daughter), or even more females, one could expect that social competence and role play would be higher developed than in families as the mother may be the only one who reacts well toward social cues in the family. A very recent study showed for instance that mothers of 4–6-year-old children saw problems for the family climate with respect to crowding in the home for children of both sexes, while they thought that too much space posed only a problem for their boys (Thornock et al., 2019). The authors of this study presume that boys would show less proximity-seeking than girls which in turn negatively affected the family climate.

In short, based on previous research about the sex-ratio in family composition, we hypothesize that in female majority families, both fathers and mothers would show a preference for social toys in the current experiment, while in male majority families, fathers with sons only would show a preference for mechanical toys, but mothers would not.

### The Current Study

In short, the current study investigates the impact of AQ and gender-balance in families on toy choice. Turner and Gervai (1995) showed that mothers who work may also show more ‘masculine’ assertive childrearing patterns. Like Turner and Gervai, we tested mothers and fathers of girls, of boys, and of girls and boys, but we also included samples of non-parents as controls.

We recruited a gender-balanced sample of parents by first asking them whether they had daughters, sons, or both sons and daughters. Hence, parents were selected based on the real situation of living in a family which was in the majority either female, or male, or rather gender-mixed. Rather than investigating whether parents had a traditional gender-specific or egalitarian attitude, we expected that parents who had a personalities with elevated AQ scores would be more inclined to select technical toys rather than educational or social role-play toys independently of their own sex, but more so if the male sex was in the majority in the family. We also recruited a control group of non-parents who were also just asked to shop toys in the current experiment. This was important as we hypothesized that the control group would only shop in accordance with their AQ score and not based on a family situation.

We developed a computerized toy choice paradigm that involves selecting either cuddly animals or role-play figures (social toys), academic, music or sports toys (educational toys) or constructive or constructed toys (technical toys). Each category had 30 exemplars so that the experiment consisted of decisions about 90 items which increased the reliability of the decision-making measurement. The aim of our study is to investigate whether high AQ parents may make different choices for their offspring than more sociable parents, in particular it is predicted that they will select more technical toys. We predicted that this effect would be maximized in fathers who live in male majority families, and attenuated in fathers who live in female majority families.

## Materials and Methods

### Participants

Originally, 234 participants were recruited. Mothers and fathers in this study took part as individuals and were from different families. Because the between-subject factors sex of parent and sex of offspring were stratified, we accordingly screened participants before the actual toy choice experiment for these variables as well as for their AQ score (see Procedure). Forty-two men and 32 females who had volunteered did not take part in the computerized toy choice task. Hence, sampling was a non-random selection process (Cook and Campbell, 1979).

The final sample size is *N* = 160, with 80 male and 80 female adult participants. Participants were well educated, 28.1% held a High School degree, 40.0% a Bachelor degree, 28.8% a Masters degree, and 3.1% a Ph.D. We did not ask for the actual profession of the parents, e.g., whether it was a technical or engineering profession. Level of education and AQ showed a non-significant negative correlation (Pearson, two-tailed) in women, *r* = −0.10, *p* = 0.352, but there was a trend for men, *r* = −0.22, *p* = 0.051, showing that the higher the educational level, the lower the AQ.

Twenty participants of either sex were parents of girls, 20 parents of boys and 20 parents of boys and girls. The mean age of both women and men was 35 years, with a range of 19–55 years for men and a range of 25–55 years for women. Age differences between men and women were not significant, *F*(1,160) = 0.118, *p* = 0.732.

Age differences between the four groups of parent status (sons only *M* = 38 years, range 26–49 years; daughters only *M* = 36 years, range 25–49 years; sons and daughters *M* = 39 years, range 26–53 years; non-parents, *M* = 30 years, range 19–55 years) were significant, though, *F*(1,160) = 14.44, *p* < 0.001, η = 0.22. *Post hoc* tests within the analysis of variance (Bonferroni corrected) showed that non-parents, although they were also in their thirties and showed a comparable age range, were on average 6–9 years younger than the three parent groups which did not significantly differ from each other in terms of age. Hence, we controlled the analyses of variance with age as covariate.

The mean AQ of the men was 20.1 with a range from 8 to 31 and a median of 21.5. The mean AQ of the women was 20.0 with a range from 4 to 30 and a median of 22.0. The four groups did not differ in AQ, *F*(3,160) = 0.58, *p* = 0.576 (non-parent *M* = 19.8, sons *M* = 20.7, daughters *M* = 20.5, sons and daughters *M* = 19.3).

### Apparatus and Material

#### AQ Questionnaire

A paper copy of the Adult Autism Spectrum Quotient (AQ) questionnaire (Baron-Cohen et al., 2001) was used to measure participants’ AQ, see also the online version on https://psychology-tools.com/test/autism-spectrum-quotient. This is a short, easy to use and easy to score tool, consists of 50 questions, and evaluates five different areas: social skill, attention switching, attention to detail, communication and imagination, with ten items contributing to each category. Internal consistency scores of this questionnaire range from 0.63 to 0.77 (Baron-Cohen et al., 2001). The questionnaire shows good test–retest reliability indicating that the items of the test are stable over time (Broadbent et al., 2013) as well as a high validity of the questionnaire as a self-report measure to quantify autistic traits (Woodbury-Smith et al., 2005). The maximum possible score was 50 points. Baron-Cohen et al. (2001) suggest that a score above 32 indicates Asperger Syndrome. A low AQ score implies that the person has a preference to socialize and agrees to statements such as ‘I find it hard to make new friends’(negative) or, I enjoy social chitchat’ (positive). A high AQ score suggests that the person is not adept in communication and shows repetitive behaviors by agreeing to statements such as, People often tell me that I keep going on and on about the same thing’ or ‘I prefer to do things the same way over and over again.’ Also other autistic traits such as high concentration ‘I frequently get so absorbed in one thing that I lose sight of other things,’ sensory sensitivity ‘I often notice small sounds when others do not’ and attention to detail ‘I tend to notice details that others do not’ are items where agreement would contribute to a higher AQ score.

#### Toy Choice Task

A Toshiba Portege R500 Windows 7 PC with a 14′′ screen and an Intel Core i5-3317U/1.70GHz Dual Core processor with a 1280 × 800 resolution LCD display at 75 MHz refresh rate was used for the toy choice paradigm. Participant responses were registered with a built-in PS/2 Microsoft mouse.

The toy choice paradigm was programed with SuperLab 5.0.1. All trials presented static pictures on a white background, see Appendix Table A1. Each category had 30 items. Stimuli belonged to three categories: (1) social toys (10 pictures of playmobil figures, 10 pictures of dolls, 10 pictures of cuddly toys), (2) educational toys (10 pictures of musical instruments, 10 pictures of sports items, 10 pictures of school items) and (3) technical toys (10 pictures of Lego items, 10 pictures of matchbox cars, 10 pictures of metal constructions). The pictures of the Playmobil figures showed various professions, all figures holding an item, e.g., a sports cup, a gun, a sword, a guitar etc. The dolls were five pictures of Barbie dolls and five action men, e.g., batman, superman, agents etc. Stimuli were presented in groups of threes, one of each corresponding category, so that participants could choose one of them. Picture size was 5.0 × 5.0 cm, with a 2.5 cm space between them. Stimuli in each trial were randomly allocated without replacement. Thus, the experiment had a total of 30 trials, presented in random order. Because the task was self-paced, stimuli were on the screen until the participant made a response. Participant responses were recorded as choice reaction time (RT) in milliseconds. Percent of toy type selection was calculated, with the total of 30 trials as 100 percent.

The initial instruction was ‘This is your shopping list for your child. Please choose only one toy that you would like to buy for your child every time by using the mouse. Pretend that you have as much money as you need, so price does not make any difference. But answer as quickly as possible, though avoid choosing randomly. Click with the mouse anywhere on the screen.’ Thereafter, on each trial, the instruction was displayed ‘Please select one toy’ above the three pictures of toys. It was clear to non-parent control participants that this was a simulated pretend situation in a toy choice experiment.

### Procedure

The study was approved by the Psychology Research Ethics Committee of the London Metropolitan University according to the Ethics Guidelines of the British Psychological Society. All participants signed an informed consent form and afterward received a debrief form both of which had been approved by the Ethics Committee. Female participants were approached by the author CS, and male participants were approached by the author MK. They were asked if they were parents, or not. If required for inclusion in the stratified sample, participants completed the AQ questionnaire. If still required for inclusion in the stratified sample, they then took part in the toy choice experiment.

### Data Generation

The Superlab 5.0.1 raw data were re-formatted with Visual Basic 6.0 and averaged per toy category using Excel and SPSS. Participants were divided into high and low AQ groups following the guidelines by Baron-Cohen et al. (2001). Scores between 0 and 21 indicate low or average autistic traits, while scores of 22 or above indicate autistic tendencies or mild autism. No participant had reached the clinical threshold score of 35. There were *n* = 40 high and *n* = 40 low AQ women, and *n* = 38 low and *n* = 42 high AQ men.

The sample size was computed based on a minimal cell frequency of five participants for the between-subject factors parent sex with two levels, children status with four levels and AQ with two levels: 2 × 4 × 2 = 16 × 5 = 80. In fact, with 160 participants we well exceeded the minimum required sample size. Repeated factors do not require additional participants because participants respond to variations of the same stimulus more than once.

## Results

Data were analyzed with a 2 (parent sex) × 4 (children status: non-parent, boys only, girls only, boy and girl) by 3 (toy choice: social, educational, technical) MANCOVA with repeated measures on the last factor and AQ score and age of parent as covariates. Nine men did not select social toys in a single trial, and two men did not select educational toys, thus for RTs, the sample is reduced by 11 participants to *N* = 149 because of missing data. If Mauchley’s Test of Sphericity was significant, degrees of freedom were adjusted according to Huynh–Feldt. Pairwise tests with Bonferroni corrections were carried out with a 95% confidence interval.

### Toy Choice

**FIGURE 1:**
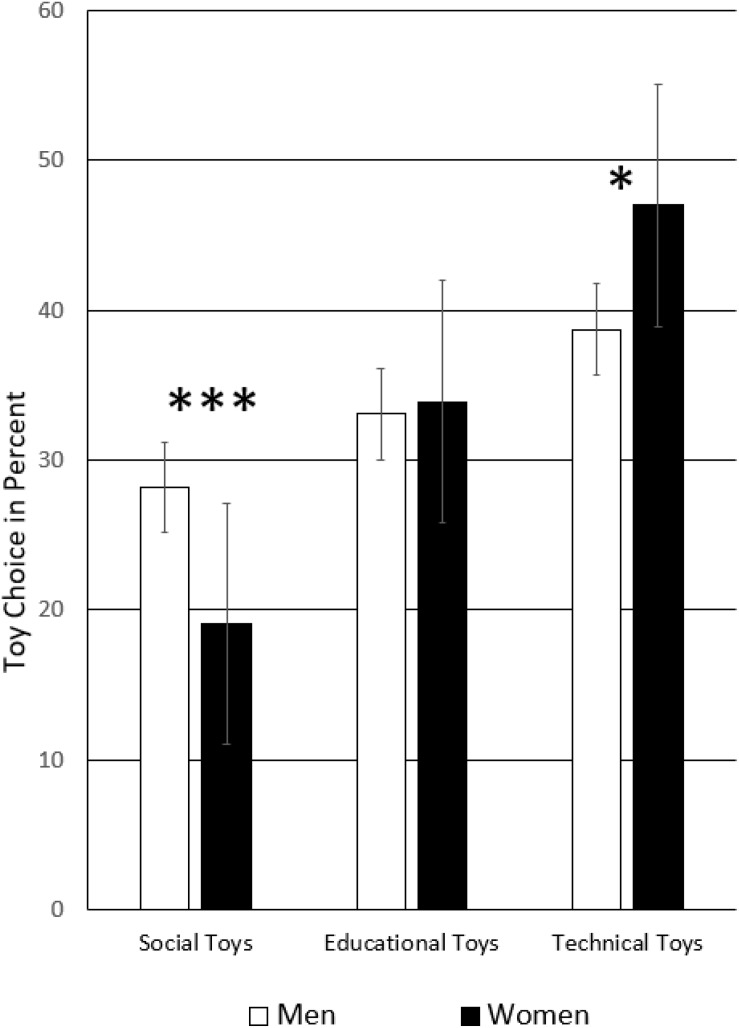
Toy selection in adult men and women. ^∗∗∗^*p* < 0.001, ^∗^*p* < 0.05.

None of the between-subject effects were significant, *p*_s_ > 0.281. However, there were several significant within-subjects interactions. The two-way interaction of parent sex by toy choice was significant, *F*(2,160) = 6.78, *p* = 0.001, η = 0.04, see Figure 1. While the trend away from social toys toward technical toys was the same in men and women, with educational toys in between, *post hoc* tests for independent samples showed that this trend was less pronounced in women because they selected significantly more social toys, *t*(158) = 3.35, *p* = 0.001, CI = [3.74, 14.50] and fewer technical toys than men, *t*(158) = −2.38, *p* = 0.018, CI = [−15.01, −1.40]. Because the overall trend is the same for either half of the sample, it is called the standard toy choice in the following text.

**FIGURE 2:**
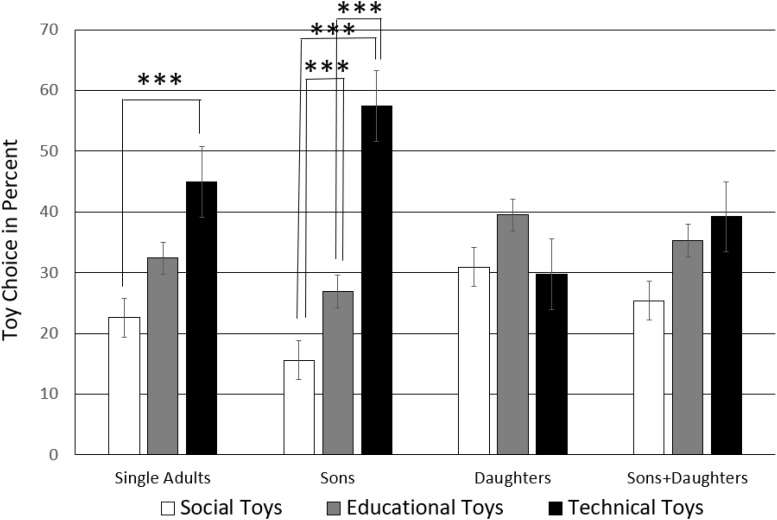
Toy selection by parents of boys only, girls only, boys and girls as well as non-parent controls. The significance threshold of the *p*-level (^∗∗∗^) is Bonferroni-adjusted to *p* = 0.05/9 = 0.006.

Toy choice also varied based on children status, *F*(6,160) = 9.07, *p* = 0.001, η = 0.15, with a larger effect size than for sex of the parent, see Figure 2. Pairwise comparisons (significance threshold of *p*-level adjusted to 0.05/9 = 0.006) showed that the non-parents selected technical toys significantly more often than social toys, *t*(39) = −3.60, *p* = 0.001, CI = [−33.18, −9.33]. The same trend showed in the case in parents of sons and daughters, but did not reach the adjusted significance threshold, *t*(39) = −2.35, *p* = 0.024, CI = [−0.24.51, −1.82]. Parents of sons selected technical toys more often than any other kind of toy (social-technical, *t*(39) = −10.11, *p* < 0.001, CI = [−51.60, −34.40]; educational-technical, *t*(39) = −6.09, *p* < 0.001, CI = [−41.63, −20.87]).

They also chose educational toys more often than social toys, *t*(39) = −3.11, *p* = 0.003, CI = [−19.38, −4.12]. Thus, parents of sons made the sharpest distinctions between toys, while parents of daughters showed no significant preference, *p*_s_ > 0.067, and did not show the standard toy choice in their selection.

**FIGURE 3:**
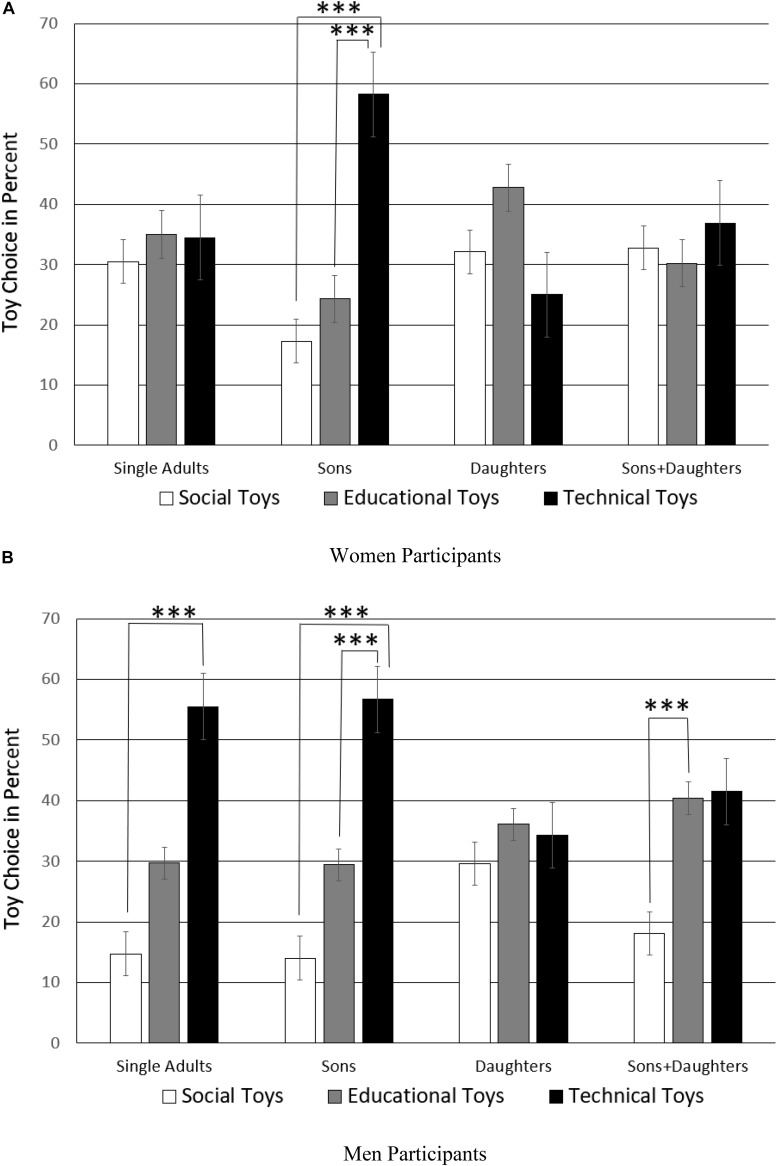
Toy selection by parents of boys only, girls only, boys and girls, and non-parents. The significance threshold of the *p*-level (^∗∗∗^) is Bonferroni-adjusted to *p* = 0.05/18 = 0.003. The plotted data are split sample means of **(A)** Women and **(B)** Men participants.

However, this selection pattern varied according to the sex of the parent, that is the three-way interaction of toy choice by children status by sex of the parent was significant, *F*(6,160) = 2.19, *p* = 0.044, η = 0.04, see Figure 3. Pairwise comparisons for the sample split by sex of the parent (significance threshold of *p*-level adjusted to 0.05/18 = 0.003) showed that females without children did not show a toy preference, *p*_s_ > 0.741 while mothers of boys show a clear preference for technical toys over both social and educational toys (social-technical, *t*(19) = −6.29, *p* < 0.001, CI = [−55.33, −27.69]; educational-technical, *t*(19) = −5.60, *p* < 0.001, CI = [−46.04, −20.98]), with no difference between social and educational toys *t*(19) = −1.49, *p* = 0.152, CI = [−19.21, 3.21]. However, this preference for mechanical toys had disappeared when mothers had only daughters, *p*_s_ > 0.018. Mothers of boys and girls did not show a preference, *p*_s_ > 0.217, just like females without children.

In contrast, different to female non-parents, male non-parents showed a clear preference for technical over social toys, *t*(19) = −5.74, *p* < 0.001, CI = [−55.27, −25.73]. This clear preference for technical toys was also present in fathers of sons-only (social-technical, *t*(19) = −8.07, *p* < 0.001, CI = [−56.04, −32.95]; educational-technical, *t*(19) = −3.42, *p* = 0.003, CI = [−46.71, −11.26]). However, this preference for technical toys was changed in fathers of daughters-only who showed no preference for any kind of toy, *p*_s_ > 0.399. Fathers of boys and girls chose social toys more often than educational toys (social-educational, *t*(19) = −3.87, *p* < 0.001, CI = [−35.69, −10.65]).

**FIGURE 4:**
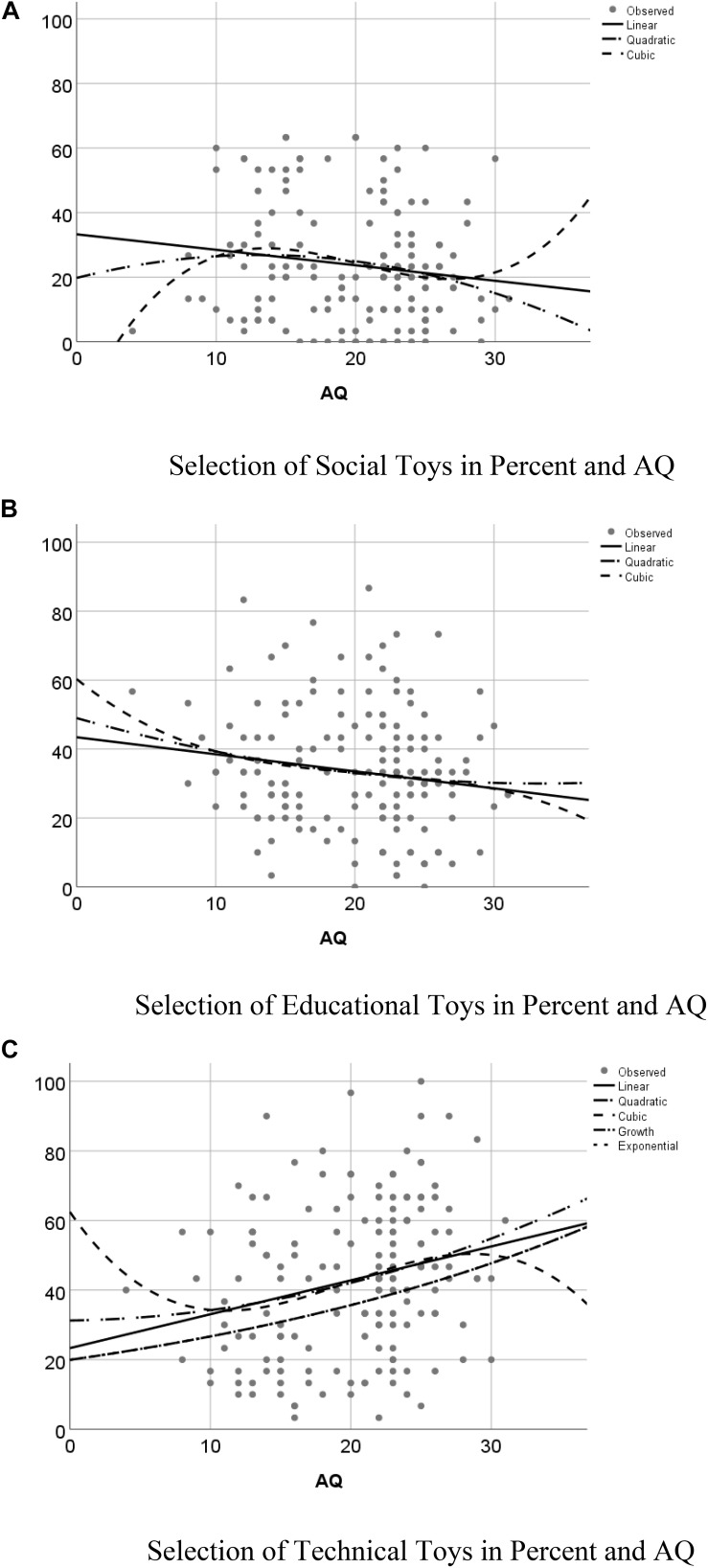
Scatterplot of the variables AQ Score and Toy selection type. The plotted data are individual participant means of toy category selection: **(A)** Social toys, **(B)** Educational toys and **(C)** Technical Toys.

Most importantly for the hypothesis that parents with a high AQ score would prefer technical toys, the two-way interaction of toy choice with AQ was significant, *F*(2,160) = 6.67, *p* = 0.001, η = 0.04. We ran Curvefit models with AQ as independent variable and the three types of toys as dependent variables, see the scatterplots in Figures 4A–C. The linear regression for social toys showed a trend, *F*(1,159) = 3.46, *p* = 0.065, which showed that the higher the AQ, the less likely was the selection of a social toy, see Figure 4A. The quadratic curvefit was not significant, *p* = 0.114, and the trend for the cubic curvefit, *F*(3,159) = 2.38, *p* = 0.072, appears to hinge on an outlier with a low AQ who rarely selected a social toy. The linear regression for educational toys was marginally significant, *F*(1,159) = 3.67, *p* = 0.057, because again, the higher the AQ, the less likely the selection of an educational toy, see Figure 4B. Both the quadratic and the cubic curvefit were not significant, *p* > 0.151. Finally, the linear regression for technical toys was significant, *F*(1,159) = 9.57, *p* = 0.002, and demonstrated that the higher the AQ, the more likely was the selection of a technical toy, see Figure 4C. Also the quadratic curvefit, *F*(2,159) = 4.87, *p* = 0.009, and the cubic curvefit, *F*(3,159) = 3.56, *p* = 0.016, were significant, but to a lesser degree.

In order to explore the role of AQ in more depth, we ran the same MANCOVA again, but with AQ as dichotomized between-subjects factor instead of as covariate (see the data generation paragraph in the Section “Materials and Methods”). Pairwise *t*-tests (two-tailed) of split samples of high and low AQ individuals (significance threshold of *p*-level adjusted to 0.05/6 = 0.008) were carried out. Individuals with a low AQ selected educational (*M* = 37.1%, *SD* = 18.5%) and technical toys (*M* = 37.7%, *SD* = 22.7%) to about the same degree, *p* = 0.892, and they selected these two more often than social toys (social-technical, *t*(77) = −2.83, *p* = 0.006, CI = [−21.12, −3.67]; social-educational, *t*(77) = −3.33, *p* = 0.001, CI = [−18.90, −4.77]).

In contrast, participants with a high AQ showed the standard toy choice (social *M* = 22.1%, *SD* = 15.1%, educational *M* = 30.1%, *SD* = 16.5%, technical *M* = 47.8%, *SD* = 20.5%) with significant distinctions between all categories (social-technical, *t*(81) = −7.26, *p* < 0.001, CI = [−32.74, −18.65]; social-educational, *t*(81) = −2.99, *p* = 0.004, CI = [−13.26, −2.67], educational-technical, *t*(81) = −4.72, *p* < 0.001, CI = [−25.19, −10.26]).

**FIGURE 5:**
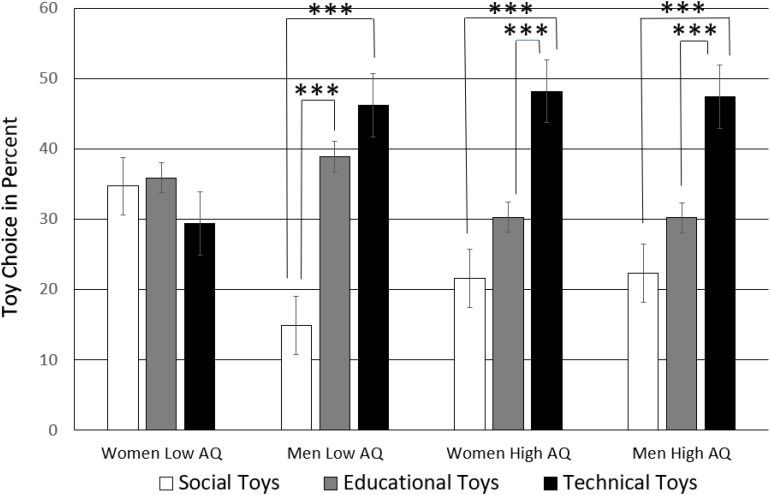
Toy selection by men and women of low and high AQ. The significance threshold of the *p*-level (^∗∗∗^) is Bonferroni-adjusted adjusted to *p* = 0.05/12 = 0.004.

This two-way interaction was qualified further as AQ interacted three-way with sex of the parent and toy choice, *F*(2,160) = 8.83, *p* = 0.001, η = 0.06, see Figure 5. Pairwise *t*-tests (two-tailed) of split samples of high and low AQ men and women (significance threshold of *p*-level adjusted to 0.05/12 = 0.004) were carried out. Low AQ individuals differed in their toy choice preferences according to their sex. The more sociable women with a low AQ had no toy preference, *p*_s_ > 0.161. The more sociable men with a low AQ to a large degree preferred educational and technical toys over social toys (social-technical, *t*(81) = −6.00, *p* < 0.001, CI = [−41.78, −20.68]; social-educational, *t*(81) = −5.36, *p* < 0.001, CI = [−32.02, −14.46]), but they made no difference between educational and technical toys, *p* = 0.244.

To directly compare low AQ men and women’s toy choice preference, independent samples *t*-tests (equal variances not assumed) were run per toy category. Low AQ women chose social toys (*M* = 34.8%, *SD* = 20.6%) more than twice as often as low AQ men (*M* = 15.2%,%, *SD* = 14.0), *t*(68.86) = 4.95, *p* < 0.001, CI = [19.65, 3.97], while low AQ men chose technical toys (*M* = 46.4%,%, *SD* = 23.1%) significantly more often than low AQ women (*M* = 29.3%,%, *SD* = 19.0%), *t*(71.72) = −3.55, *p* = 0.001, CI = [−17.07, 4.80]. There was no sex difference between low AQ men and women with regards to educational toys, *p* = 0.543.

In contrast, high AQ individuals showed the standard toy choice preference whether they were male or female. Figure 5 shows that the toy choice pattern was nearly identical in individuals with high AQ as if this personality trait was overriding their sex. There was a small statistical difference, though, because of the differently sized standard deviations. High AQ women distinguished technical toys (social-technical, *t*(39) = −5.77, *p* < 0.001, CI = [−35.91, −17.26]; educational-technical, *t*(39) = −3.72, *p* = 0.001, CI = [−27.54, −8.19]) and so did high AQ men who also selected technical more often than social, (*t*(41) = −4.61, *p* < 0.001, CI = [−35.72, −13.96]), and educational toys (*t*(41) = −3.04, *p* = 0.004, CI = [−29.20, −5.87]).

**FIGURE 6:**
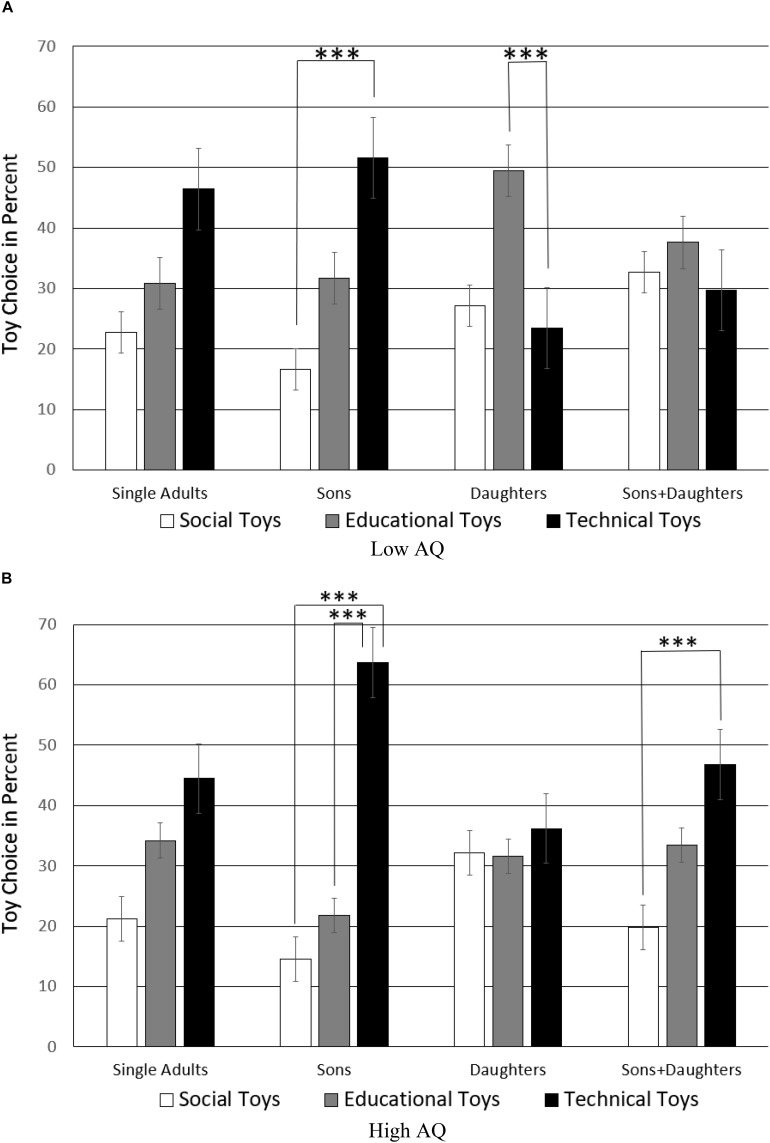
Toy selection by low and high AQ parents depending on their children’s sex. The significance threshold of the *p*-level (^∗∗∗^) is Bonferroni-adjusted adjusted to *p* = 0.05/24 = 0.002. The plotted data are split sample means of participants with **(A)** low AQ and with **(B)** high AQ.

AQ was also significant in interaction with toy choice by child status, *F*(6,160) = 2.43, *p* = 0.026, η = 0.05, see Figure 6. Pairwise *t*-tests (two-tailed) of split samples of gender family type individuals with high and low AQ (significance threshold of *p*-level adjusted to 0.05/24 = 0.002) were carried out. Non-parents had a similar standard toy preference whether their AQ was high or low, but these differences did not reach the Bonferroni adjusted *p*-level, *p*_s_ > 0.011. Low AQ parents of boys showed a preference for technical over social toys *t*(18) = −5.02, *p* < 0.001, CI = [−50.28, −20.60], but if parents had a high AQ, this maximized technical toy choice for boys (social-technical, *t*(20) = −10.71, *p* < 0.001, CI = [−59.54, −40.14]; educational-technical, *t*(20) = −6.91, *p* < 0.001, CI = [−54.13, −29.04]). While more sociable parents of daughters showed a significant preference for educational toys, *t*(17) = 4.59, *p* < 0.001, CI = [14.42, 38.94], high AQ parents of daughters appeared to select at random, *p*_s_ > 0.398. The more sociable low AQ parents of boys and girls showed no toy preference, *p*_s_ > 0.197, while high AQ parents of boys and girls preferred technical over social toys (social-technical, *t*(18) = −4.84, *p* < 0.001, CI = [−41.51, −16.39]), with other differences not reaching the Bonferroni adjusted *p*-level, *p*_s_ > 0.004. These results about the impact of the mere presence of sons only, daughters only, or both sons and daughters, occurred independently of the sex of the parents as the four-way interaction of AQ, toy choice, sex of parents, and children status was not significant, *F*(6,160) = 0.923, *p* = 0.478. This means that there is an effect of parents’ AQ on toy choice depending on whether they are male or female, but a second independent effect of parents’ AQ based on the sex of the children they happened to conceive.

### Choice Reaction Time

As mentioned before, nine men had not selected a social toy on a single occasion, and two men had not selected an educational toy which reduced the sample size to *N* = 149. The same MANCOVA was run for choice RTs as for choice in percent. Not a single within-subjects factor reached significance, *p*_s_ > 0.070. However, the sex of the parents was significant, *F*(1,149) = 47.24, *p* < 0.001, η = 0.25. Men decided in about two thirds of the time (*M* = 2728 ms, *SE* = 153.99) compared to women (*M* = 4287 ms, *SE* = 166.50). Moreover, there was a main effect of AQ, *F*(1,149) = 10.96, *p* = 0.001, η = 0.07. When this model was re-run with AQ high/low scores as a between-subjects factor instead of as a covariate, it turned out that more sociable individuals decided slower (*M* = 3909 ms, *SE* = 165.46) than high AQ individuals (*M* = 3163 ms, *SE* = 160.01) and this occurred independently of their sex, *F*(1,149) = 2.01, *p* = 0.159.

## Discussion

The current forced-choice study for the first time demonstrates that AQ of the parents shapes their toy choice as well as parents’ mere exposure to offspring of one-sex-only. We called the preference sequence of social to educational toys to technical toys the ‘standard toy choice’ because both women and men showed the same pattern, it was just more pronounced in men but not different (see Figure 1). The stratified sample according to the factor sociability (high/low AQ) of parents and gender majority in the family (sex of own children) allowed us to test for deviations from this pattern. We found that boys-only families and parents’ high AQ maximize their technical toy choice. In contrast, in daughters-only families, technical toy choice was depressed as shown by either no preference, or selection of educational toys. The study also produced further additional evidence for the recent finding that people with high AQ make more consistent decisions than low AQ individuals (Farmer et al., 2017): High AQ parents of either sex showed a nearly identical toy choice pattern.

The general toy choice pattern that emerged was that there was a more or less pronounced trend that adults selected social toys the least often, educational toys such as academic and sports items more often, and technical toys the most. We called this priority the standard toy choice pattern because it showed in both men and women – parent or not – and in men in an even more pronounced fashion than in women. This result confirmed our expectation that life-like figures and cuddly toys would be less interesting (Crichton and Lange-Küttner, 1999; Servin et al., 1999).

There were no sex differences in toy choice of high AQ parents, both sexes showed this ‘standard toy preference’ in a nearly identical fashion. This result is in complete accordance with recently published research showing that both clinically autistic and high AQ adults make more consistent choices between two items in repeated presentations when a third decoy item varies (Farmer et al., 2017). Farmer et al. explain that this consistent judgment would be an advantage as neurotypical adults would usually be influenced by attractive lures in legal judgments, elections, consumer behavior etc. However, consistent decision-making would be a disadvantage if unexpected circumstances would need to be considered. In the current toy choice task, the decisions of high AQ parents were in complete agreement with the majority of the participants. However, the control for AQ also showed that while low AQ men had the same standard toy choice preference as high AQ individuals, low AQ women were the only group who did not show this preference. This gives some support to the autism theory of the extreme male brain whereby men are likely to show the same autistic traits just more or less pronounced (Baron-Cohen, 2002). However, if women had a high AQ score, they showed the same toy preferences as high AQ men.

It would be interesting to find out in follow-up research whether low AQ men and women are more likely to be homemakers and housekeepers who are not competing on the labor market, or whether they would work in more social professions. A limitation of the current study is that we did not ask for the specific profession of the parents.

Farmer et al. (2017) also investigated whether the more consistent choices that high AQ individuals make would result from slower, more deliberate decision making, but they did not find a significant difference in the non-clinical AQ sample. In the current study, the low AQ participants did decide slower than high AQ individuals, and women slower than men, but the two factors did not significantly interact with each other. Women did select more social and fewer technical toys than men but this was not mirrored in RTs as no interactions were found between RTs, sex of parents and toy choice.

Moreover, daughters had an impact on toy choice, especially when they did not have brothers, that is, when there was no boy in the family. This situation where the father was the only male in the family modified the standard toy choice in men, even if they had a high AQ. Moreover, the impact of daughters-only families occurred in high AQ individuals independently of their sex, that is in both high AQ men and women. Daughters-only families also had an impact on low AQ individuals whose preference was reversed in comparison to those with sons-only – educational toys such as academic, music and sports items were preferred significantly more often than technical toys. Hence, a predominantly female environment with the father as the only man in the family had the power to change the otherwise quite robust standard toy choice. However, as soon as a son was in the family in addition to a daughter, the standard toy choice showed again in high AQ individuals, and sons-only families maximized their technical toy choice. Thus, we conclude an involuntary and non-controllable factor such as the sex of their offspring may bias parents’ toy choice toward technical toys in addition to their own frame of mind.

Also earlier research found a three-way interaction between the sex of the parent, the sex of the child and the toy category (Campenni, 1999). Campenni had also categorized parents according to whether they had girls only, boys only, or both boys and girls. Mothers and fathers did not differ in their ratings of gender-neutral toys. Mothers also showed less stereotype and were gender-neutral in their ratings of ‘male’ toys. However, fathers who had girls only rated ‘feminine’ toys such as a vacuum cleaner as more gender-stereotyped than mothers of girls. One might wonder about this result given that vacuum cleaners are also used to clean cars and are quite heavy technical gear. In contrast, fathers of boys and girls were the most gender neutral in their ratings of both kinds of gender-typed toys.

The current study used a quasi-experimental stratified sample of parents with a high or low AQ. The scores were not in the clinical spectrum and thus should more be seen as an indication of a personality trait that shows how social parents were, and how much they were interested in technical and systematic thought. As such, neither personality feature is positive or negative. Previous research showed that as soon as infants can reach for toys at 4–5 months of age, they prefer mechanically moving toys because they are more predictable than live animals and more interesting than cuddly toys (Crichton and Lange-Küttner, 1999). Moreover, recent research showed no looking preferences for sex-specific toys and faces in 4- and 5-months old infants (Escudero et al., 2013), thus, these authors suggested that there is no innate sex-related toy choice bias in the infants themselves. Accordingly, parents may set up their nursery in a way which is in tune with their own sociability (Boe and Woods, 2018). The current study adds to this evidence and shows that toy choice is determined by both the personality traits of parents and the gender distribution in the entire family.

## Data Availability

The datasets generated for this study are available on request to the corresponding author.

## Ethics Statement

This study was approved by the Psychology Research Ethics Committee of the London Metropolitan University according to the Ethics Guidelines of the British Psychological Society. All participants signed an informed consent form and afterward received a debrief form both of which had been approved by the Ethics Committee.

## Author Contributions

All authors listed have made a substantial, direct and intellectual contribution to the work, and approved it for publication.

## Conflict of Interest Statement

The authors declare that the research was conducted in the absence of any commercial or financial relationships that could be construed as a potential conflict of interest.

## Appendix

**TABLE A1:** Stimulus Material (randomized sequence in the experiment).
